# Supplementary material for: Genome-wide identification of PP2A gene family in Camellia sinensis reveals the potential role of CsPP2A-TON2/FASS1 in abiotic stress
Source: PeerJ. 2025 May 27;13:e19431. doi: 10.7717/peerj.19431 (PMC12124296; doi:10.7717/peerj.19431)
Supplement: Supplemental Information 1 [file peerj-13-19431-s001.docx]

**Genome-wide identification of PP2A gene family in *Camellia sinensis* reveals the**

**potential role of *CsPP2A-TON2/FASS1* in abiotic stress**

Surjit Bhattacharjee^1,4#^, Abhirup Paul^1#^, Aradhana Jana^1#^, G Meher Unnati^1#^, Deepak R^1^, Ye Miao^2^, Lu Hongling^3^, Guoxin Shen^3*^, Neelam Mishra^4*^

^1^Independent researcher

Bengaluru, Karnataka,

India

^2^Qingtian Forestry Technology Extended Station,

Qingtian, China

^3^Zhejiang Academy of Agricultural Sciences

Hangzhou, China

^4^Department of Botany

St. Joseph’s University, Bengaluru, Karnataka,

India

#These authors contributed equally to this work.

*Corresponding authors:

Guoxin Shen, Ph.D., Professor, Tel: +86-571-86404298; Fax: +86-571-86404298

Email address: [guoxin.shen@ttu.edu](mailto:guoxin.shen@ttu.edu)

Neelam Mishra, Ph.D., Assistant professor

Email address: neelamiitkgp@gmail.com

; neelammishra@sju.edu.in

**Supplementary File 1**

**Table S1. BLAST positives table for PP2A gene family in *C. sinensis*.** The table shows both the A and B subfamilies. The PP2A protein sequences of *Arabidopsis thaliana* were used as query sequences to search for their homologs in tea database.

| **Arabidopsis PP2A gene IDs** | **Tea PP2A gene IDs** | **Identity percentage** | **Positive percentage** | **Gap percentage** |
| --- | --- | --- | --- | --- |
| **A subfamily** | | | | |
| AtPP2A-A1 (AT1G25490) | TEA002042.1 | 66% | 75% | 14% |
| AtPP2A-A2 (AT3G25800) | TEA002042.1 | 71% | 76% | 14% |
|  | TEA011483.1 | 67% | 74% | 15% |
| AtPP2A-A3 (AT1G13320) | TEA002042.1 | 70% | 76% | 14% |
|  | TEA011483.1 | 66% | 74% | 14% |
| **B subfamily** | | | | |
| AtPP2ABBα (AT1G51690) | TEA015525.1 | 77% | 84% | 6% |
| AtPP2ABBβ (AT1G17720) | TEA015525.1 | 77% | 84% | 8% |
| AtPP2AB'α (AT5G03470) | TEA021355.1 | 69% | 84% | 2% |
|  | TEA019300.1 | 65% | 80% | 1% |
| AtPP2AB'β (AT3G09880) | TEA021355.1 | 71% | 83% | 2% |
|  | TEA019300.1 | 69% | 83% | 0% |
| AtPP2AB′γ (AT4G15415) | TEA015045.1 | 70% | 78% | 9% |
|  | TEA019300.1 | 69% | 79% | 6% |
| AtPP2AB′δ (AT3G26030) | TEA019300.1 | 69% | 83% | 1% |
|  | TEA015045.1 | 55% | 70% | 14% |
| At PP2AB′ε (AT3G54930) | TEA021355.1 | 61% | 78% | 3% |
|  | TEA019300.1 | 62% | 79% | 0% |
| AtPP2AB′ζ (AT3G54930) | TEA015045.1 | 70% | 80% | 6% |
| AtPP2AB′η (AT3G26020) | TEA019300.1 | 79% | 88% | 0% |
|  | TEA015045.1 | 66% | 75% | 8% |
| AtPP2AB′θ (AT1G13460) | TEA019300.1 | 75% | 86% | 2% |
|  | TEA015045.1 | 64% | 74% | 10% |
| AtPP2AB′κ (AT5G25510) | TEA009324.1 | 71% | 85% | 1% |
|  | TEA000454.1 | 60% | 74% | 9% |
| At PP2AB′′α (AT5G44090) | TEA000364.1 | 74% | 84% | 1% |
|  | TEA021728.1 | 69% | 81% | 3% |

| AtPP2AB′′β (AT5G28850) | TEA000364.1 | 78% | 87% | 1% |
| --- | --- | --- | --- | --- |
|  | TEA021728.1 | 74% | 84% | 4% |
| AtPP2AB′′γ (AT5G28900) | TEA000364.1 | 72% | 85% | 2% |
|  | TEA021728.1 | 75% | 84% | 3% |
| AtPP2AB′′δ (AT1G54450) | TEA000364.1 | 75% | 85% | 1% |
|  | TEA021728.1 | 76% | 84% | 4% |
| AtPP2AB′′ε (AT1G03960) | TEA000364.1 | 75% | 85% | 1% |
|  | TEA021728.1 | 76% | 84% | 4% |
| At PP2A-TON2/FASS1 (AT5G18580) | TEA018948.1 | 79% | 82% | 13% |

Table S2. Table showing the list of all the cis-acting elements (CAREs) identified from 2 kbp upstream region of all the identified PP2A (A and B subfamily) genes of *C. sinensis.*

| **Sl.**  **no.** | **Cis-regulatory element identified** | **Sequence** | **Sequence length** | **Tea genes** | **Specific function of the cis-regulatory element** |
| --- | --- | --- | --- | --- | --- |
| 1. | AACA-motif | TAACAAACTCCA | 12 | TEA002042.1 | Endosperm-specific negative expression |
| 2. | ABRE | ACGTG/ CACGTG/ GCAACGTGTC | 5/ 6/ 10 | TEA002042.1/ TEA011483.1/ TEA015525.1/ TEA019300.1/ TEA015045.1/ TEA000454.1/  TEA000364.1/ TEA018948.1 | Abscisic acid responsiveness |
| 3. | Box 4 | ATTAAT | 6 | TEA002042.1/ TEA011483.1/ TEA015525.1/ TEA021355.1/ TEA019300.1/ TEA015045.1/ TEA009324.1/ TEA000454.1/ TEA000364.1/ TEA018948.1 | Conserved DNA module involved in light responsiveness |
| 4. | CAT-box | GCCACT | 6 | TEA002042.1/  TEA000364.1/ TEA018948.1 | Meristem expression |
| 5. | G-box | CACGTC/ CACGTGAAA/ TAAACGTG/ TCCACATGGCA | 6/ 8/ 9/ 10 | TEA011483.1/ TEA015525.1/ TEA019300.1/ TEA015045.1/ TEA000454.1/  TEA000364.1/ TEA018948.1 | Light responsiveness |
| 6. | GCN4-motif | TGAGTCA | 7 | TEA002042.1/ TEA019300.1 | Endosperm expression |
| 7. | GT1-motif | GGTTAAT/ GGTTAA | 6/ 7 | TEA002042.1/ TEA011483.1/ TEA021355.1/ TEA019300.1/  TEA015045.1/ | Light responsiveness |

|  |  |  |  | TEA009324.1/ TEA018948.1 |  |
| --- | --- | --- | --- | --- | --- |
| 8. | LAMP-element | CTTTATCA | 8 | TEA002042.1 | Light responsiveness |
| 9. | LTR | CCGAAA | 6 | TEA002042.1/ TEA011483.1/ TEA015045.1/  TEA021728.1/ TEA018948.1 | Low-temperature responsiveness |
| 10. | TCCC-motif | TCTCCCT | 7 | TEA002042.1/ TEA015525.1/ TEA015045.1/  TEA000364.1/ TEA018948.1 | Light responsiveness |
| 11. | 3-AF1 binding site | TAAGAGAGGAA | 11 | TEA011483.1 | Light responsiveness |
| 12. | AE-box | AGAAACAA | 8 | TEA011483.1/ TEA021355.1/ TEA019300.1 | Light responsiveness |
| 13. | ARE | AAACCA | 6 | TEA011483.1/ TEA015525.1/ TEA021355.1/ TEA019300.1/ TEA015045.1/ TEA009324.1/ TEA021728.1/  TEA018948.1 | Regulatory element essential for the anaerobic induction |
| 14. | AT-rich sequence | TAAAATACT | 9 | TEA011483.1 | Maximal elicitor-mediated activation |
| 15. | AT1-motif | AATTATTTTTTATT | 14 | TEA011483.1 | Light responsiveness |
| 16. | CGTCA-motif | CGTCA | 5 | TEA011483.1/ TEA015525.1/ TEA021355.1/ TEA019300.1/ TEA015045.1/ TEA009324.1/ TEA000454.1/ TEA000364.1/ TEA021728.1/ TEA018948.1 | MeJA-responsiveness |
| 17. | MBS | CAACTG | 6 | TEA011483.1/  TEA019300.1/ TEA015045.1 | Drought-inducibility |
| 18. | O2-site | GTTGACGTGA/ GATGATGTGG | 10 | TEA011483.1/ TEA015525.1/ TEA021355.1/  TEA015045.1/ TEA018948.1 | Regulatory element involved in zein metabolism regulation |
| 19. | P-box | CCTTTTG | 7 | TEA011483.1/ TEA019300.1/ TEA009324.1 | Gibberellin-responsive element |
| 20. | TCA-element | CCATCTTTTT/ TCAGAAGAGG | 10 | TEA011483.1/ TEA019300.1/ TEA009324.1/ TEA000454.1/  TEA000364.1 | Salicylic acid responsiveness |
| 21. | TCT-motif | TCTTCA | 6 | TEA011483.1/ TEA009324.1/  TEA000454.1/ TEA000364.1 | Light responsive element |
| 22. | TGACG-motif | TGACG | 5 | TEA011483.1/ TEA015525.1/ TEA021355.1/ TEA019300.1/ TEA015045.1/  TEA009324.1/ TEA000454.1/ | MeJA-responsiveness |

|  |  |  |  | TEA000364.1/ TEA021728.1/  TEA018948.1 |  |
| --- | --- | --- | --- | --- | --- |
| 23. | AT-rich element | ATAGAAATCAA | 11 | TEA015525.1/ TEA000454.1/ TEA021728.1 | binding site of AT-rich DNA binding protein |
| 24. | Box II | ACACGTAGA | 9 | TEA015525.1 | Light responsive element |
| 25. | HD-Zip 3 | GTAAT(G/C)ATTAC | 11 | TEA015525.1 | Protein binding site |
| 26. | MBSI | AAAAAAC(G/C)GTTA | 12 | TEA015525.1/ TEA019300.1/ TEA018948.1 | Involved in flavonoid biosynthetic genes regulation |
| 27. | TC-rich repeats | ATTCTCTAAC | 10 | TEA015525.1/ TEA021355.1/ TEA018948.1 | Involved in defense and stress responsiveness |
| 28. | GATA-motif | AAGGATAAGG/ AAGATAAGATT/ GATAGGA | 7/ 10/ 11 | TEA021355.1/ TEA019300.1/ TEA009324.1/  TEA000364.1 | Light responsive element |
| 29. | Gap-box | CAAATGAA(A/G)A | 10 | TEA021355.1 | Light responsive element |
| 30. | RY-element | CATGCATG | 8 | TEA021355.1/ TEA019300.1 | Regulatory element involved in seed-specific regulation |
| 31. | TGA-element | AACGAC | 6 | TEA021355.1/ TEA019300.1 | Auxin responsive element |
| 32. | CCAAT-box | CAACGG | 6 | TEA019300.1/ TEA015045.1/ TEA018948.1 | MYBHv1 binding site |
| 33. | I-box | CGATAAGGCG | 10 | TEA019300.1 | Light responsive element |
| 34. | ACE | GACACGTATG | 10 | TEA015045.1 | Light responsive element |
| 35. | ATCT-motif | AATCTAATCC | 10 | TEA015045.1/  TEA009324.1/ TEA000364.1 | Light responsive element |
| 36. | GA-motif | ATAGATAA | 8 | TEA015045.1 | Light responsive element |
| 37. | MSA-like element | TCCAACGGT | 9 | TEA015045.1 | Cell cycle regulation |
| 38. | GARE-motif | TCTGTTG | 7 | TEA009324.1 | Gibberellin responsive element |
| 39. | HD-Zip 1 | CAAT(A/T)ATTG | 9 | TEA009324.1 | Differentiation of the palisade mesophyll cells |
| 40. | MRE | AACCTAA | 7 | TEA009324.1/  TEA000364.1/ TEA018948.1 | Light responsive element |
| 41. | Sp1 | GGGCGG | 6 | TEA009324.1/ TEA000364.1 | Light responsive element |
| 42. | chs-CMA1a | TTACTTAA | 8 | TEA009324.1/ TEA018948.1 | Light responsive element |
| 43. | Chs-CMA2a | TCACTTGA | 8 | TEA000454.1 | Light responsive element |
| 44. | GC-motif | CCCCCG | 6 | TEA000364.1 | Anoxic specific inducibility |
| 45. | Box III | ATCATTTTCACT | 12 | TEA018948.1 | Protein binding site |

**Table S3: GO enrichment analysis of the PP2A genes (A and B subfamilies) of *C. sinensis*.** The potential functions of the PP2A genes have been classified in 3 major categories based on their biological function, cellular component and molecular function.

| **B I O L O G I C A L P R O C E S S** | | | | |
| --- | --- | --- | --- | --- |
| **GO Ids** | **Description** | **Frequency of genes** | **Genes** | **References** |
| GO:0051225 | Spindle assembly | 1 | TEA019300.1 | Matsuoka et al., 2007; Binns et al., 2009 |
| GO:0007165 | Signal transduction | 2 | TEA015045.1/ TEA009324.1 | Ruiz-Gómez et al., 1997; Binns et al., 2009 |
| GO:0023052 | Signaling | 2 | TEA015045.1/ TEA009324.1 | Kido et al., 2002; Zhang et al., 2013; Binns et al., 2009 |
| GO:0000913 | Preprophase band assembly | 1 | TEA018948.1 | Binns et al., 2009 |
| GO:0000226 | Microtubule cytoskeleton organisation | 1 | TEA018948.1 | Subramanian et al., 2010; Binns et al., 2009 |
| GO:0030865 | Cortical cytoskeleton organisation | 1 | TEA018948.1 | Binns et al., 2009 |
| **C E L L U L A R C O M P O N E N T** | | | | |
| **GO Ids** | **Description** | **Number of**  **genes** | **Genes** | **References** |
| GO:0000159 | Protein phosphatase type 2A complex | 3 | TEA015525.1/ TEA015045.1/ TEA009324.1 | Rossio et al., 2013; McCright and Virshup, 1995; Binns et al., 2009 |
| GO:0070652 | HAUS complex | 1 | TEA019300.1 | Lawo et al., 2009; Binns et al., 2009 |
| GO:0008287 | Protein serine/threonine phosphatase complex | 2 | TEA015045.1/ TEA009324.1 | Binns et al., 2009 |
| GO:0005634 | Nucleus | 1 | TEA018948.1 | Baskaran and Rao, 1991; Binns et al., 2009 |
| GO:0009524 | Phragmoplast | 1 | TEA018948.1 | Binns et al., 2009 |
| GO:0005813 | Centrosome | 1 | TEA018948.1 | Jakobsen et al., 2011; Binns et al., 2009 |
| GO:0005819 | Spindle | 1 | TEA018948.1 | Özlü et al., 2010; Binns et al., 2009 |
| **M O L E C U L A R F U N C T I O N** | | | | |
| **GO Ids** | **Description** | **Number of genes** | **Genes** | **References** |
| GO:0008601 | Protein phosphatase type 2A regulator activity | 2 | TEA015525.1/ TEA009324.1 | Rossio et al., 2013; McCright and Virshup, 1995; Binns et al., 2009 |
| GO:0019888 | Protein phosphatase regulator activity | 2 | TEA015045.1/ TEA009324.1 | Rossio et al., 2013; McCright and Virshup, 1995; Binns et al., 2009 |
| GO:0046872 | Metal ion binding | 2 | TEA000364.1/ TEA018948.1 | Gugnoni et al., 2017; Brown, 2005; Binns et al., 2009 |
| GO:0005509 | Calcium ion binding | 1 | TEA018948.11 | Bumba et al., 2016; Binns et al., 2009 |

**Table S4: RMSD (root mean square deviation) score values of superimposed arabidopsis and tea plant PP2A proteins.** The superimposed protein pairs with lowest RMSD scores are colour coded (blue depicting arabidopsis proteins and green depicting tea proteins).

| **Arabidopsis PP2A gene IDs** | **Tea PP2A gene IDs** | **RMSD scores** |
| --- | --- | --- |
| **A subfamily** | | |
| AtPP2A-A1 (AT1G25490) | TEA002042.1 (CsPP2A-A1) | 2.784 |
|  | TEA011483.1 (CsPP2A-A2) | 2.090 |
| AtPP2A-A2 (AT3G25800) | TEA002042.1 (CsPP2A-A1) | **0.009** |
|  | TEA011483.1 (CsPP2A-A2) | 0.624 |
| AtPP2A-A3 (AT1G13320) | TEA002042.1 (CsPP2A-A1) | 0.035 |
|  | TEA011483.1 (CsPP2A-A2) | **0.616** |
| **B subfamily** | | |
| AtPP2ABBα (AT1G51690) | TEA015525.1 (CsPP2A-B1) | 0.452 |
| AtPP2ABBβ (AT1G17720) | TEA015525.1 (CsPP2A-B1) | **0.303** |
| AtPP2AB'α (AT5G03470) | TEA021355.1 (CsPP2A-B2) | 0.337 |
|  | TEA019300.1 (CsPP2A-B3) | 0.619 |
| AtPP2AB'β (AT3G09880) | TEA021355.1 (CsPP2A-B2) | **0.300** |
|  | TEA019300.1 (CsPP2A-B3) | **0.492** |
| AtPP2AB′γ (AT4G15415) | TEA015045.1 (CsPP2A-B4) | 0.372 |
|  | TEA019300.1 (CsPP2A-B3) | 0.588 |
| AtPP2AB′δ (AT3G26030) | TEA019300.1 (CsPP2A-B3) | 0.609 |
|  | TEA015045.1 (CsPP2A-B4) | 0.533 |

| AtPP2AB′ε (AT3G54930) | TEA021355.1 (CsPP2A-B2) | 0.509 |
| --- | --- | --- |
|  | TEA019300.1 (CsPP2A-B3) | 0.597 |
| AtPP2AB′ζ (AT3G54930) | TEA015045.1 (CsPP2A-B4) | 0.383 |
| AtPP2AB′η (AT3G26020) | TEA019300.1 (CsPP2A-B3) | 0.474 |
|  | TEA015045.1 (CsPP2A-B4) | 0.390 |
| AtPP2AB′θ (AT1G13460) | TEA019300.1 (CsPP2A-B3) | 0.510 |
|  | TEA015045.1 (CsPP2A-B4) | **0.367** |
| AtPP2AB′κ (AT5G25510) | TEA009324.1 (CsPP2A-B5) | **0.214** |
|  | TEA000454.1 (CsPP2A-B6) | **0.558** |
| AtPP2AB′′α (AT5G44090) | TEA000364.1 (CsPP2A-B7) | 18.511 |
|  | TEA021728.1 (CsPP2A-B8) | 0.373 |
| AtPP2AB′′β (AT5G28850) | TEA000364.1 (CsPP2A-B7) | 18.486 |
|  | TEA021728.1 (CsPP2A-B8) | **0.327** |
| AtPP2AB′′γ (AT5G28900) | TEA000364.1 (CsPP2A-B7) | 18.521 |
|  | TEA021728.1 (CsPP2A-B8) | 0.497 |
| AtPP2AB′′δ (AT1G54450) | TEA000364.1 (CsPP2A-B7) | 18.401 |
|  | TEA021728.1 (CsPP2A-B8) | 0.529 |
| AtPP2AB′′ε (AT1G03960) | TEA000364.1 (CsPP2A-B7) | 18.401 |
|  | TEA021728.1 (CsPP2A-B8) | 0.529 |
| AtPP2A-TON2/FASS1 (AT5G18580) | TEA018948.1 (CsPP2A-B9) | **0.428** |
| **CsPP2A-B7 superimposed with all AtPP2A proteins** | | |
| TEA000364.1 (CsPP2A-B7) | AtPP2A-A1 (AT1G25490) | 11.707 |
|  | AtPP2A-A2 (AT3G25800) | 17.0 |
|  | AtPP2A-A3 (AT1G13320) | 12.052 |
|  | AtPP2ABBα (AT1G51690) | 17.109 |
|  | AtPP2ABBβ (AT1G17720) | 17.304 |
|  | AtPP2AB'α (AT5G03470) | 20.492 |
|  | AtPP2AB'β (AT3G09880) | 20.676 |
|  | AtPP2AB′γ (AT4G15415) | 17.492 |
|  | AtPP2AB′δ (AT3G26030) | 22.138 |
|  | AtPP2AB′ε (AT3G54930) | 16.561 |
|  | AtPP2AB′ζ (AT3G54930) | 16.818 |
|  | AtPP2AB′η (AT3G26020) | 20.207 |
|  | AtPP2AB′θ (AT1G13460) | 21.040 |
|  | AtPP2AB′κ (AT5G25510) | 20.979 |
|  | AtPP2A-TON2/FASS1 (AT5G18580) | 17.333 |

Table S5: Z-scores of the predicted PP2A protein structures of arabidopsis and tea plant.

| **Sl.No.** | **Arabidopsis** | **Z-score** |
| --- | --- | --- |
| Sub-unit A | | |
| 1 | AtPP2AA1 | -14.65 |
| 2 | AtPP2AA2 | -14.51 |
| 3 | AtPP2AA3 | -14.26 |
| Sub-unit B | | |
| 1 | AtPP2ABBα | -7.42 |
| 2 | AtPP2ABBβ | -6.93 |
| 3 | AtPP2AB'α | -10.12 |
| 4 | AtPP2AB'β | -10.36 |
| 5 | AtPP2AB′γ | -9.51 |
| 6 | AtPP2AB′δ | -9.98 |
| 7 | AtPP2AB′ε | -9.42 |
| 8 | AtPP2AB′ζ | -9.25 |
| 9 | AtPP2AB′η | -9.41 |
| 10 | AtPP2AB′θ | -10.04 |
| 11 | AtPP2AB′κ | -10.09 |
| 12 | AtPP2AB′′α | -10.6 |
| 13 | AtPP2AB′′β | -9.04 |
| 14 | AtPP2AB′′γ | -9.93 |
| 15 | AtPP2AB′′δ | -9.68 |

| 16 | AtPP2AB′′ε | -9.74 |
| --- | --- | --- |
| 17 | AtPP2A-TON2-FASS1 | -9.35 |
| **Sl.No.** | **Tea** | **Z-score** |
| Sub-unit A | | |
| 1 | CsPP2AA1 | -12.33 |
| 2 | CsPP2AA2 | -12.49 |
| Sub-unit B | | |
| 1 | CsPP2AB1 | -5.62 |
| 2 | CsPP2AB2 | -9.8 |
| 3 | CsPP2AB3 | -9.77 |
| 4 | CsPP2AB4 | -9.06 |
| 5 | CsPP2AB5 | -10.2 |
| 6 | CsPP2AB6 | -9.47 |
| 7 | CsPP2AB7 | -3.35 |
| 8 | CsPP2AB8 | -8.48 |
| 9 | CsPP2AB9 | -7.54 |

Table S6. Tissue specific expression levels of all the tea PP2A (A and B subfamilies) genes. The expressionvalues have been given in transcripts per million (TPM).

| **Gene** | **Apical bud** | **Flower** | **Fruit** | **Young leaf** | **Mature leaf** | **Old leaf** | **Root** | **Stem** |
| --- | --- | --- | --- | --- | --- | --- | --- | --- |
| **TEA002042.1** (*CsPP2A-A1*) | 47.21572779 | 40.80571594 | 31.76685047 | 41.69425108 | 35.81367386 | 27.40336594 | 37.71095723 | 42.74203902 |
| **TEA011483.1** (*CsPP2A-A2*) | 24.62665564 | 22.19557771 | 17.60154028 | 26.43921163 | 28.91821916 | 25.25322891 | 23.80559258 | 28.26086708 |
| **TEA015525.1** (*CsPP2A-B1/ CsPP2AB55α*) | 37.04537571 | 47.20326481 | 33.85966082 | 31.24712013 | 34.92274028 | 33.11385135 | 30.15113927 | 44.75998601 |
| **TEA021355.1** (*CsPP2A-B2/*  *CsPP2AB'α*) | 9.529215895 | 18.42935977 | 19.34707205 | 7.627519724 | 16.05536558 | 18.28051731 | 25.69554707 | 13.72203951 |
| **TEA019300.1** (*CsPP2A-B3/*  *CsPP2AB'β*) | 16.14257955 | 18.78087345 | 12.88586282 | 16.79768389 | 17.86507442 | 15.03354923 | 13.13175634 | 27.08853597 |
| **TEA015045.1** (*CsPP2A-B4/*  *CsPP2AB′γ*) | 15.52779143 | 11.1078321 | 13.68094797 | 15.5378576 | 13.12270921 | 13.16197246 | 8.362803812 | 14.47156267 |
| **TEA009324.1** (*CsPP2A-B5/*  *CsPP2AB′δ*) | 60.67958767 | 33.52436126 | 28.33062039 | 47.45345698 | 25.79995163 | 23.96488769 | 22.10169579 | 42.31923108 |
| **TEA000454.1** (*CsPP2A-B6/*  *CsPP2AB′ε*) | 34.05047929 | 8.817971597 | 28.09300873 | 27.63047595 | 25.37304596 | 10.93349035 | 14.56146284 | 53.76387328 |
| **TEA000364.1** (*CsPP2A-B7/*  *CsPP2AB′′α*) | 10.61826914 | 10.46506424 | 16.20328708 | 9.855783914 | 25.59577935 | 19.15972314 | 10.26255081 | 18.09425798 |
| **TEA021728.1** (*CsPP2A-B8/*  *CsPP2AB′′β*) | 9.265735271 | 14.12080645 | 6.269292124 | 7.901767625 | 7.860632745 | 8.896518422 | 6.658907016 | 11.02183425 |
| **TEA018948.1** (*CsPP2A-B9/*  *CsPP2A-TON2/FASS1*) | 68.58400638 | 37.60191988 | 50.24572609 | 56.64933189 | 58.38399126 | 28.53501701 | 38.60207567 | 77.48916427 |

Table S7. Expression levels of all the tea PP2A (A and B subfamilies) genes under cold stress. The expression values have been given in transcripts per million (TPM).

| **Gene** | **CK** | **CA1-6h** | **CA1-7d** | **CA2-7d** | **DA-7d** |
| --- | --- | --- | --- | --- | --- |
| **TEA002042.1** (*CsPP2A-A1*) | 14.94166702 | 14.98687437 | 24.65551908 | 20.48601978 | 22.50249662 |
| **TEA011483.1** (*CsPP2A-A2*) | 12.0135845 | 16.95642297 | 34.57665424 | 32.35170119 | 31.64737073 |
| **TEA015525.1** (*CsPP2A-B1/ CsPP2AB55α*) | 25.91937849 | 36.74039526 | 33.38519604 | 31.34630639 | 40.98103367 |
| **TEA021355.1** (*CsPP2A-B2/*  *CsPP2AB'α*) | 6.716083372 | 9.135039656 | 8.736038605 | 10.16537437 | 8.474511159 |
| **TEA019300.1** (*CsPP2A-B3/*  *CsPP2AB'β*) | 11.83924386 | 11.61672681 | 11.82388573 | 12.59277818 | 20.26149549 |
| **TEA015045.1** (*CsPP2A-B4/*  *CsPP2AB′γ*) | 8.169191029 | 10.14048911 | 10.89530963 | 9.231106787 | 10.62905125 |
| **TEA009324.1** (*CsPP2A-B5/*  *CsPP2AB′δ*) | 15.08151541 | 16.70445591 | 19.71885706 | 14.91196819 | 22.78843744 |
| **TEA000454.1** (*CsPP2A-B6/*  *CsPP2AB′ε*) | 17.39514944 | 12.95545831 | 18.55729727 | 14.24543308 | 30.27790932 |
| **TEA000364.1** (*CsPP2A-B7/*  *CsPP2AB′′α*) | 16.14767971 | 22.25696882 | 29.90380946 | 27.53449178 | 20.09900982 |
| **TEA021728.1** (*CsPP2A-B8/*  *CsPP2AB′′β*) | 3.8381915 | 5.872838043 | 7.513193665 | 8.553197029 | 7.955886338 |
| **TEA018948.1** (*CsPP2A-B9/*  *CsPP2A-TON2/FASS1*) | 37.05429502 | 34.15958648 | 48.10258924 | 38.75172329 | 37.91671528 |

| **Gene** | **N-0h** | **PEG-N-24h** | **PEG-N-48h** | **PEG-N-72h** |
| --- | --- | --- | --- | --- |
| **TEA002042.1** (*CsPP2A-A1*) | 15.65475514 | 15.3922017 | 17.31353401 | 10.6092825 |
| **TEA011483.1** (*CsPP2A-A2*) | 20.44895222 | 29.38649816 | 28.19320673 | 29.54603352 |
| **TEA015525.1** (*CsPP2A-B1/ CsPP2AB55α*) | 21.70161935 | 22.17854927 | 27.21974434 | 29.76187135 |
| **TEA021355.1** (*CsPP2A-B2/*  *CsPP2AB'α*) | 23.48729362 | 16.47441788 | 14.74138142 | 10.44243976 |
| **TEA019300.1** (*CsPP2A-B3/*  *CsPP2AB'β*) | 9.430656737 | 7.864161184 | 9.22253428 | 8.00933972 |
| **TEA015045.1** (*CsPP2A-B4/*  *CsPP2AB′γ*) | 7.94179204 | 5.628877946 | 8.384088712 | 6.938951726 |
| **TEA009324.1** (*CsPP2A-B5/*  *CsPP2AB′δ*) | 15.49992789 | 9.076555023 | 11.39668463 | 6.259356932 |
| **TEA000454.1** (*CsPP2A-B6/*  *CsPP2AB′ε*) | 11.67024722 | 3.327453586 | 4.487936175 | 2.953864933 |
| **TEA000364.1** (*CsPP2A-B7/*  *CsPP2AB′′α*) | 8.085322634 | 7.261598955 | 9.326169493 | 5.434333542 |
| **TEA021728.1** (*CsPP2A-B8/*  *CsPP2AB′′β*) | 2.16848464 | 1.252870294 | 1.845972174 | 0.922720193 |
| **TEA018948.1** (*CsPP2A-B9/*  *CsPP2A-TON2/FASS1*) | 44.07960441 | 34.0285492 | 33.33523421 | 24.44984591 |

Table S8. Expression levels of all the tea PP2A (A and B subfamilies) genes under drought stress. The expression values have been given in transcripts per million (TPM).

Table S9. Expression levels of all the tea PP2A (A and B subfamilies) genes under salt stress. The expression values have been given in transcripts per million (TPM).

| **Gene** | N-0h | NaCl-N-24h | NaCl-N-48h | NaCl-N-72h |
| --- | --- | --- | --- | --- |
| **TEA002042.1** (*CsPP2A-A1*) | 15.6548114 | 12.53771727 | 22.22134624 | 13.3017552 |
| **TEA011483.1** (*CsPP2A-A2*) | 20.44888353 | 20.05054861 | 29.93686394 | 20.75280972 |
| **TEA015525.1** (*CsPP2A-B1/ CsPP2AB55α*) | 21.70167614 | 22.61683709 | 21.6520741 | 21.55674018 |
| **TEA021355.1** (*CsPP2A-B2/*  *CsPP2AB'α*) | 23.4872405 | 13.29511559 | 14.99865167 | 10.34026131 |
| **TEA019300.1** (*CsPP2A-B3/*  *CsPP2AB'β*) | 9.430656994 | 5.721107879 | 6.768927465 | 7.832598855 |
| **TEA015045.1** (*CsPP2A-B4/*  *CsPP2AB′γ*) | 7.941793644 | 7.702377388 | 7.004938982 | 9.571082648 |
| **TEA009324.1** (*CsPP2A-B5/*  *CsPP2AB′δ*) | 15.5000324 | 8.064401012 | 9.957025196 | 9.683604233 |
| **TEA000454.1** (*CsPP2A-B6/*  *CsPP2AB′ε*) | 11.67024947 | 3.891342593 | 4.473769137 | 4.840359748 |
| **TEA000364.1** (*CsPP2A-B7/*  *CsPP2AB′′α*) | 8.085331877 | 9.403392329 | 7.439085003 | 5.55347162 |
| **TEA021728.1** (*CsPP2A-B8/*  *CsPP2AB′′β*) | 2.168478586 | 1.219126403 | 1.265975855 | 0.376279266 |
| **TEA018948.1** (*CsPP2A-B9/*  *CsPP2A-TON2/FASS1*) | 44.07954578 | 42.21157141 | 36.80374791 | 32.64214068 |

Table S10. Expression levels of all the tea PP2A (A and B subfamilies) genes under MeJA hormonal treatment. The expression values have been given in transcripts per million (TPM).

| **Gene** | CK | 12h_MeJA | 24h_MeJA | 48h_MeJA |
| --- | --- | --- | --- | --- |
| **TEA002042.1** (*CsPP2A-A1*) | 47.02620531 | 34.32444171 | 36.15884391 | 36.88570569 |
| **TEA011483.1** (*CsPP2A-A2*) | 49.36381488 | 35.04229683 | 34.88926124 | 35.5003364 |
| **TEA015525.1** (*CsPP2A-B1/ CsPP2AB55α*) | 33.32017551 | 24.93300914 | 23.4555809 | 25.51736133 |
| **TEA021355.1** (*CsPP2A-B2/*  *CsPP2AB'α*) | 7.177103943 | 4.718190447 | 5.369504128 | 9.189417609 |
| **TEA019300.1** (*CsPP2A-B3/*  *CsPP2AB'β*) | 22.16350853 | 18.36343527 | 17.42011619 | 18.19315429 |
| **TEA015045.1** (*CsPP2A-B4/*  *CsPP2AB′γ*) | 17.24607163 | 10.48931855 | 10.04656705 | 11.90360326 |
| **TEA009324.1** (*CsPP2A-B5/*  *CsPP2AB′δ*) | 25.45051186 | 20.84969736 | 18.1574396 | 20.12688119 |
| **TEA000454.1** (*CsPP2A-B6/*  *CsPP2AB′ε*) | 35.19282684 | 31.01342393 | 26.41231077 | 35.27980128 |
| **TEA000364.1** (*CsPP2A-B7/*  *CsPP2AB′′α*) | 17.98121505 | 12.67507272 | 12.45995889 | 16.76208854 |
| **TEA021728.1** (*CsPP2A-B8/*  *CsPP2AB′′β*) | 9.409586 | 7.492451367 | 6.948283688 | 6.267398179 |
| **TEA018948.1** (*CsPP2A-B9/*  *CsPP2A-TON2/FASS1*) | 58.14941128 | 47.0949077 | 43.38020194 | 62.60472752 |

**Table S11: Primers of PP2A genes of tea plant.**

| **S. No.** | **Gene ID** | **Forward primer** | **Reverse primer** |
| --- | --- | --- | --- |
| 1 | TEA021355.1 *(CsPP2A-B2)* | TTCCAGGTAGCAGAACGAGC | ATTGGCAGTCAGCCCATGAA |
| 2 | TEA021728.1 *(CsPP2A-B8)* | CGACCACCCCATTGACTACA | CGGTGTGGAACGAAAAGACG |
| 3 | TEA018948.1 *(CsPP2A-B9)* | AGAAGGTTCCATCAGTCACAGG | AGTGAAACCTTTTCAGCACCAG |

**Table S12: Relative expression levels of tea PP2A genes obtained through qRT-PCR.**

| **Stress induced** | | **Highly Upregulated** | **Relatively expression level** | **Downregulated** | **Relatively expression level** |
| --- | --- | --- | --- | --- | --- |
| **Cold Stress**  **(4 degrees for 2-7 days)** | 0 | TEA018948.1  *(CsPP2A-B9)* | 1 | TEA021728.1  *(CsPP2A-B8)* | 1 |
|  | 2d |  | 8.674  8.421  7.592 |  | 0.561  0.476  0.488 |
|  | 7d |  | 10.567  8.991  9.214 |  | 0.341  0.346  0.299 |
| **Salt stress**  **(200mM NaCl for 0-72 hours)** | 0 | TEA018948.1  *(CsPP2A-B9)* | 1 | TEA021728.1  *(CsPP2A-B8)* | 1 |
|  | 24h |  | 3.871  4.441  3.992 |  | 0.654  0.542  0.588 |
|  | 48h |  | 5.804  4.851  5.866 |  | 0.501  0.435  0.425 |
|  | 72h |  | 6.027  4.984  6.551 |  | 0.486  0.419  0.467 |
| **Drought stress**  **(25%PEG for 48 and 72 hours)** | 0 | TEA018948.1  *(CsPP2A-B9)* | 1 | TEA021728.1  *(CsPP2A-B8)* | 1 |
|  | 48h |  | 6.591  5.375  6.513 |  | 0.445  0.401  0.394 |
|  | 72h |  | 4.454  3.992  3.800 |  | 0.5614  0.464  0.500 |
| **Methyl Jasmonate**  **(MeJA for 12, 24, and 48 hours)** | 0 | TEA018948.1  *(CsPP2A-B9)* | 1 | TEA021355.1  *(CsPP2A-B2)* | 1 |
|  | 12h |  | 0.567  0.602  0.649 |  | 0.412  0.452  0.476 |
|  | 24h |  | 0.787  0.481  0.523 |  | 0.545  0.489  0.458 |
|  | 48h |  | 4.674  4.652  3.925 |  | 0.586  0.652  0.621 |

**Reference:**

- Baskaran, R. and Rao, M.R.S., 1991. Mammalian spermatid specific protein, TP2, is a zinc metalloprotein with two finger motifs. *Biochemical and biophysical research communications*, *179*(3), pp.1491-1499.
- Binns, D., Dimmer, E., Huntley, R., Barrell, D., O'donovan, C. and Apweiler, R., 2009. QuickGO: a web-based tool for Gene Ontology searching. *Bioinformatics*, *25*(22), pp.3045-3046.
- Brown, R.S., 2005. Zinc finger proteins: getting a grip on RNA. Current opinion in structural biology, 15(1), pp.94-98.
- Bumba, L., Masin, J., Macek, P., Wald, T., Motlova, L., Bibova, I., Klimova, N., Bednarova, L., Veverka, V., Kachala, M. and Svergun, D.I., 2016. Calcium-driven folding of RTX domain β-rolls ratchets translocation of RTX proteins through type I secretion ducts. *Molecular cell*, *62*(1), pp.47-62.
- Gugnoni, M., Sancisi, V., Gandolfi, G., Manzotti, G., Ragazzi, M., Giordano, D., Tamagnini, I., Tigano, M., Frasoldati, A., Piana, S. and Ciarrocchi, A., 2017. Cadherin-6 promotes EMT and cancer metastasis by restraining autophagy. Oncogene, 36(5), pp.667-677.
- Jakobsen, L., Vanselow, K., Skogs, M., Toyoda, Y., Lundberg, E., Poser, I., Falkenby, L.G., Bennetzen, M., Westendorf, J., Nigg, E.A. and Uhlen, M., 2011. Novel asymmetrically localizing components of human centrosomes identified by complementary proteomics methods. The EMBO journal, 30(8), pp.1520-1535.
- Kido, M., Shima, F., Satoh, T., Asato, T., Kariya, K.I. and Kataoka, T., 2002. Critical Function of the Ras-associating Domain as a Primary Ras-binding Site for Regulation of Saccharomyces cerevisiaeAdenylyl Cyclase. Journal of Biological Chemistry, 277(5), pp.3117-3123.
- Lawo, S., Bashkurov, M., Mullin, M., Ferreria, M.G., Kittler, R., Habermann, B., Tagliaferro, A., Poser, I., Hutchins, J.R., Hegemann, B. and Pinchev, D., 2009. HAUS, the 8-subunit human Augmin complex, regulates centrosome and spindle integrity. Current biology, 19(10), pp.816-826.
- Matsuoka, S., Ballif, B.A., Smogorzewska, A., McDonald III, E.R., Hurov, K.E., Luo, J., Bakalarski, C.E., Zhao, Z., Solimini, N., Lerenthal, Y. and Shiloh, Y., 2007. ATM and ATR substrate analysis reveals extensive protein networks responsive to DNA damage. science, 316(5828), pp.1160-1166.
- McCright, B. and Virshup, D.M., 1995. Identification of a new family of protein phosphatase 2A regulatory subunits (∗). Journal of Biological Chemistry, 270(44), pp.26123-26128.
- Özlü, N., Monigatti, F., Renard, B.Y., Field, C.M., Steen, H., Mitchison, T.J. and Steen, J.J., 2010. Binding partner switching on microtubules and aurora-B in the mitosis to cytokinesis transition. Molecular & cellular proteomics, 9(2), pp.336-350.
- Rossio, V., Michimoto, T., Sasaki, T., Ohbayashi, I., Kikuchi, Y. and Yoshida, S., 2013. Nuclear PP2A-Cdc55 prevents APC-Cdc20 activation during the spindle assembly checkpoint. Journal of cell science, 126(19), pp.4396-4405.
- Ruiz-Gómez, A. and Mayor, F., 1997. β-Adrenergic receptor kinase (GRK2) colocalizes with β-adrenergic receptors during agonist-induced receptor internalization. *Journal of Biological Chemistry*, *272*(15), pp.9601-9604.
- Subramanian, R., Wilson-Kubalek, E.M., Arthur, C.P., Bick, M.J., Campbell, E.A., Darst, S.A., Milligan, R.A. and Kapoor, T.M., 2010. Insights into antiparallel microtubule crosslinking by PRC1, a conserved nonmotor microtubule binding protein. Cell, 142(3), pp.433-443.
