# Supplementary material for: Genome-wide identification of PP2A gene family in Camellia sinensis reveals the potential role of CsPP2A-TON2/FASS1 in abiotic stress
Source: PeerJ. 2025 May 27;13:e19431. doi: 10.7717/peerj.19431 (PMC12124296; doi:10.7717/peerj.19431)
Supplement: Supplemental Information 2 [file peerj-13-19431-s002.docx]

**Genome-wide identification of PP2A gene family in *Camellia sinensis* reveals the potential role of *CsPP2A-TON2/FASS1* in abiotic stress**

Surjit Bhattacharjee^1,4#^, Abhirup Paul^1#^, Aradhana Jana^1#^, G Meher Unnati^1#^, Deepak R^1^, Ye Miao^2^, Lu Hongling^3^, Guoxin Shen^3*^, Neelam Mishra^4*^

^1^Independent researcher Bengaluru, Karnataka, India

^2^Qingtian Forestry Technology Extended Station,

Qingtian, China

^3^Zhejiang Academy of Agricultural Sciences

Hangzhou, China

^4^Department of Botany

St. Joseph’s University, Bengaluru, Karnataka,

India

#These authors contributed equally to this work.

*Corresponding authors:

Guoxin Shen, Ph.D., Professor, Tel: +86-571-86404298; Fax: +86-571-86404298

Email address: [guoxin.shen@ttu.edu](mailto:guoxin.shen@ttu.edu)

Neelam Mishra, Ph.D., Assistant professor

Email address: neelamiitkgp@gmail.com; neelammishra@sju.edu.in

**Supplementary File 2**

**Figure S1: Transmembrane helices of PP2A genes in *C. sinensis.*** The transmembrane helices were predicted using TMHMM server v2.0 with default parameters.

# >Tea PP2A-A:

| # **TEA002042.1** Length: 513 |  | # **TEA011483.1** Length: 513 |  |
| --- | --- | --- | --- |
| # TEA002042.1 Number of predicted TMHs: 0 |  | # TEA011483.1 Number of predicted TMHs: 0 |  |
| # TEA002042.1 Exp number of AAs in TMHs: 0.43865 |  | # TEA011483.1 Exp number of AAs in TMHs: 0.04335 |  |
| # TEA002042.1 Exp number, first 60 AAs: 0 |  | # TEA011483.1 Exp number, first 60 AAs: 0 |  |
| # TEA002042.1 Total prob of N-in: 0.00085 |  | # TEA011483.1 Total prob of N-in: 0.00092 |  |
| TEA002042.1 TMHMM2.0 outside 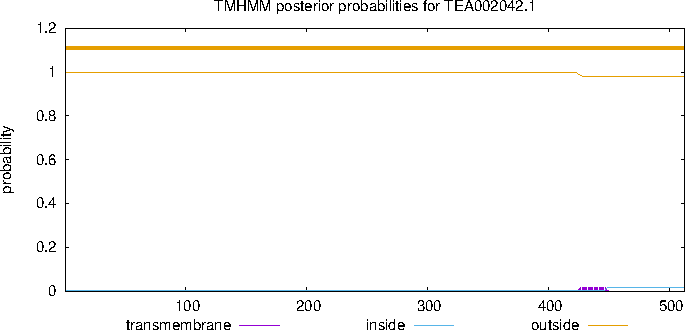 | 1 513 | TEA011483.1 TMHMM2.0 outside 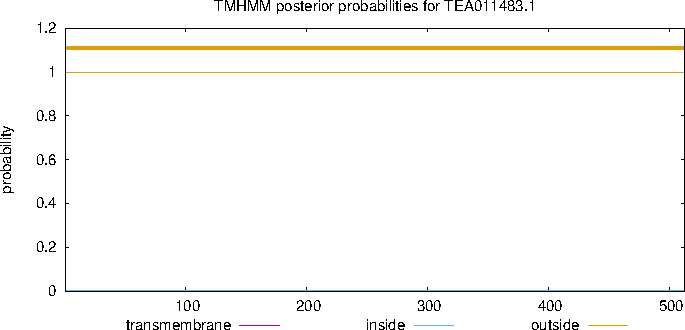 | 1 513 |
|  |  |  |  |
| **>Tea PP2A-B:** |  |  |  |
| # **TEA015525.1** Length: 522  # TEA015525.1 Number of predicted TMHs: 0  # TEA015525.1 Exp number of AAs in TMHs: 0.04028 # TEA015525.1 Exp number, first 60 AAs: 0  # TEA015525.1 Total prob of N-in: 0.00100  TEA015525.1 TMHMM2.0 outside 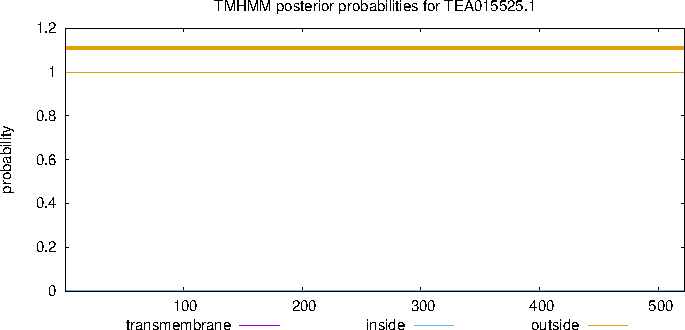 | 1 522 | # **TEA021355.1** Length: 487  # TEA021355.1 Number of predicted TMHs: 0  # TEA021355.1 Exp number of AAs in TMHs: 0.02755 # TEA021355.1 Exp number, first 60 AAs: 0.00079  # TEA021355.1 Total prob of N-in: 0.00566  TEA021355.1 TMHMM2.0 outside 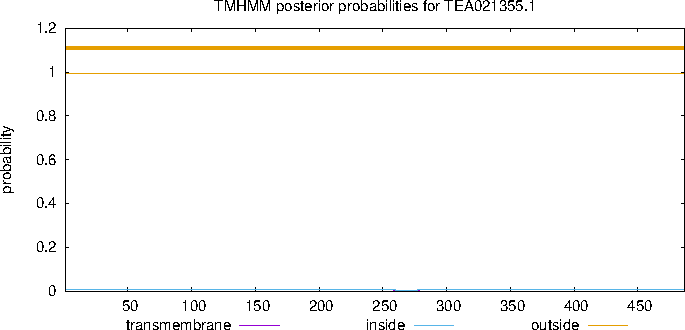 | 1 487 |
|  |  |  |  |
| # **TEA019300.1** Length: 1268 | | # **TEA015045.1** Length: 570 |  |
| # TEA019300.1 Number of predicted TMHs: 0 | | # TEA015045.1 Number of predicted TMHs: 0 |  |
| # TEA019300.1 Exp number of AAs in TMHs: 0.261070000000001 | | # TEA015045.1 Exp number of AAs in TMHs: 4.00243 |  |
| # TEA019300.1 Exp number, first 60 AAs: 0.25743 | | # TEA015045.1 Exp number, first 60 AAs: 0 |  |
| # TEA019300.1 Total prob of N-in: 0.01193 | | # TEA015045.1 Total prob of N-in: 0.00749 |  |
| TEA019300.1 TMHMM2.0 outside 1 1268 | | TEA015045.1 TMHMM2.0 outside 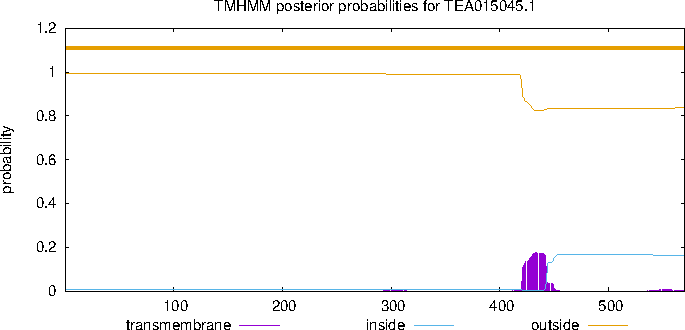 | 1 570 |
| 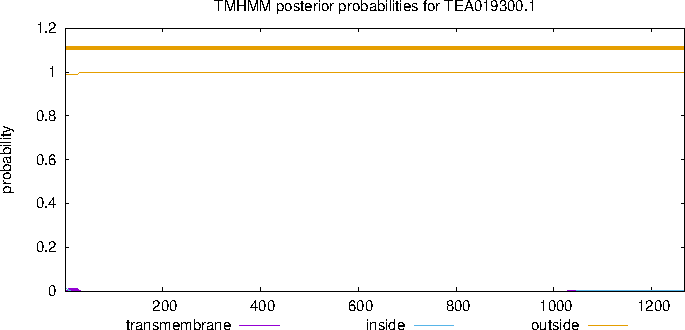 | |  |  |

# **TEA009324.1** Length: 505

# TEA009324.1 Number of predicted TMHs: 0

# TEA009324.1 Exp number of AAs in TMHs: 0.01218 # TEA009324.1 Exp number, first 60 AAs: 0.00025

# TEA009324.1 Total prob of N-in: 0.00067

TEA009324.1 TMHMM2.0 outside 1 505

# **TEA000454.1** Length: 583

# TEA000454.1 Number of predicted TMHs: 0

# TEA000454.1 Exp number of AAs in TMHs: 0.12753 # TEA000454.1 Exp number, first 60 AAs: 0.04416

# TEA000454.1 Total prob of N-in: 0.00602

TEA000454.1 TMHMM2.0 outside 1 583

| 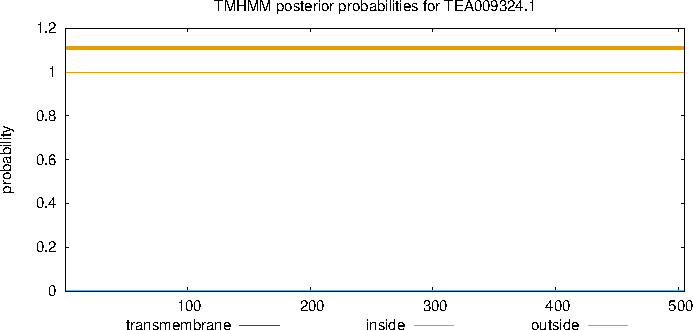 | 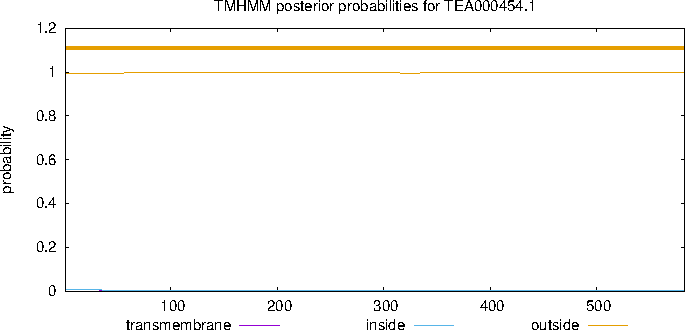 |
| --- | --- |
| # **TEA000364.1** Length: 534  # TEA000364.1 Number of predicted TMHs: 0  # TEA000364.1 Exp number of AAs in TMHs: 0.00474 # TEA000364.1 Exp number, first 60 AAs: 0  # TEA000364.1 Total prob of N-in: 0.00200  TEA000364.1 TMHMM2.0 outside 1 534  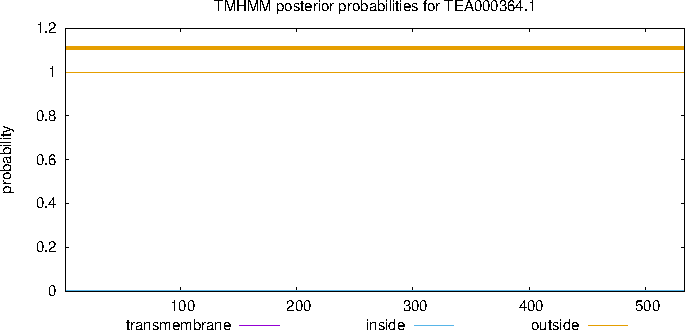 | # **TEA021728.1** Length: 718  # TEA021728.1 Number of predicted TMHs: 0  # TEA021728.1 Exp number of AAs in TMHs: 0.00321 # TEA021728.1 Exp number, first 60 AAs: 0  # TEA021728.1 Total prob of N-in: 0.00013  TEA021728.1 TMHMM2.0 outside 1 718  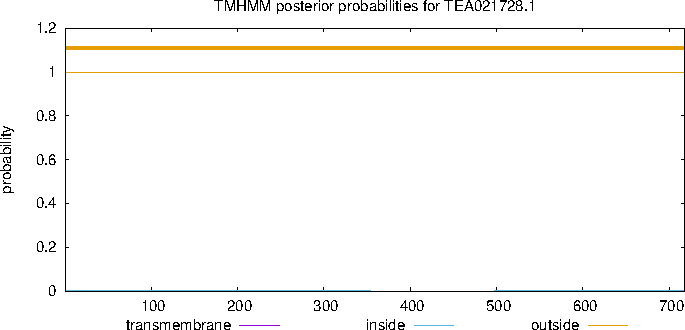 |

# **TEA018948.1** Length: 433

# TEA018948.1 Number of predicted TMHs: 0

# TEA018948.1 Exp number of AAs in TMHs: 0.00557 # TEA018948.1 Exp number, first 60 AAs: 0.00094

# TEA018948.1 Total prob of N-in: 0.00187

TEA018948.1 TMHMM2.0 outside 1 433


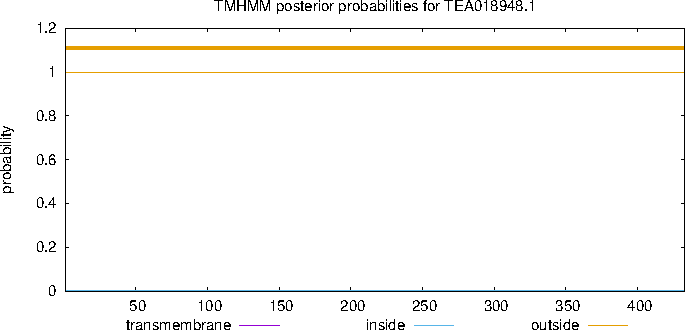


**Figure S2. The scaffold distribution of PP2A genes in tea plant.** The 11 PP2A genes were mapped onto their respective scaffolds using MapGene2chromosome web v2 (MG2C) tool (http://mg2c.iask.in/mg2c_v2.1/). The scaffolds are drawn to scale and the scaffold numbers are indicated on the top

**Figure S3. Functional interaction of PP2A-TON2/FASS1.** Protein-protein functional interactions of PP2A-TON2/FASS1, labeled in red, with other PP2A Proteins. The interaction network was constructed using the Arabidopsis PP2A-TON2/FASS1 protein. Pink lines depicted experimentally validated protein-protein interactions.


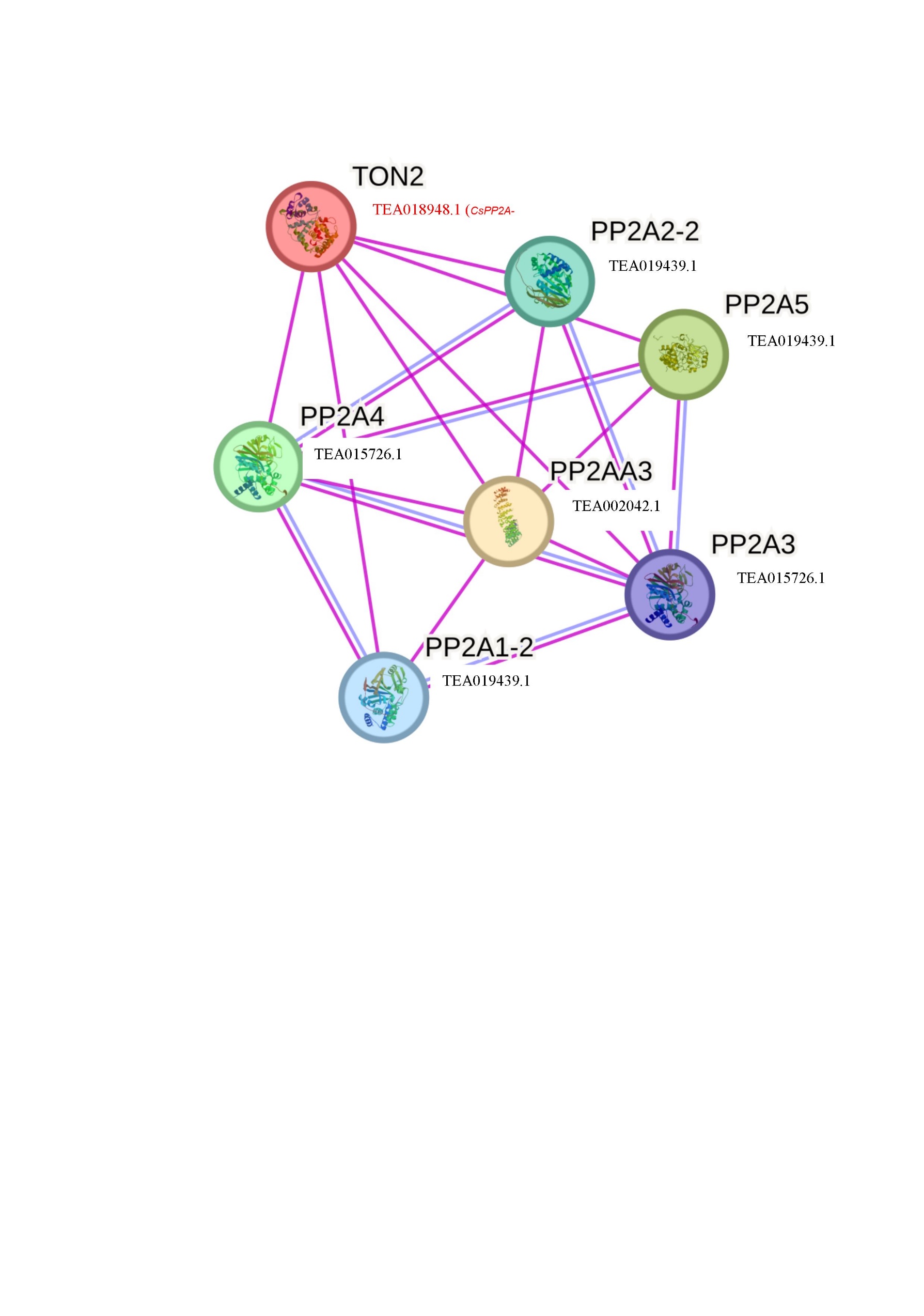


TEA018948.1 (*CsPP2A-TON2/FASS1*)
